# Increased glutamate synaptic transmission in the nucleus raphe magnus neurons from morphine-tolerant rats

**DOI:** 10.1186/1744-8069-1-7

**Published:** 2005-02-09

**Authors:** Bihua Bie, Zhizhong Z Pan

**Affiliations:** 1 Department of Anesthesiology, Unit 110, The University of Texas-MD Anderson Cancer Center, 1515 Holcombe Boulevard, Houston, Texas, 77030, USA; 2 Department of Biochemistry & Molecular Biology, The University of Texas-MD Anderson Cancer Center, 1515 Holcombe Boulevard, Houston, Texas, 77030, USA

## Abstract

Currently, opioid-based drugs are the most effective pain relievers that are widely used in the treatment of pain. However, the analgesic efficacy of opioids is significantly limited by the development of tolerance after repeated opioid administration. Glutamate receptors have been reported to critically participate in the development and maintenance of opioid tolerance, but the underlying mechanisms remain unclear. Using whole-cell voltage-clamp recordings in brainstem slices, the present study investigated chronic morphine-induced adaptations in glutamatergic synaptic transmission in neurons of the nucleus raphe magnus (NRM), a key supraspinal relay for pain modulation and opioid analgesia. Chronic morphine significantly increased glutamate synaptic transmission exclusively in one class of NRM cells that contains μ-opioid receptors in a morphine-tolerant state. The adenylyl cyclase activator forskolin and the cAMP analog 8-bromo-cAMP mimicked the chronic morphine effect in control neurons and their potency in enhancing the glutamate synaptic current was significantly increased in neurons from morphine-tolerant rats. MDL12330a, an adenylyl cyclase inhibitor, and H89, a protein kinase A (PKA) inhibitor, reversed the increase in glutamate synaptic transmission induced by chronic morphine. In addition, PMA, a phorbol ester activator of protein kinase C (PKC), also showed an increased potency in enhancing the glutamate synaptic current in these morphine-tolerant cells. The PKC inhibitor GF109203X attenuated the chronic morphine effect. Taken together, these results suggest that chronic morphine increases presynaptic glutamate release in μ receptor-containing NRM neurons in a morphine-tolerant state, and that the increased glutamate synaptic transmission appears to involve an upregulation of both the cAMP/PKA pathway and the PKC pathway. This glutamate-mediated activation of these NRM neurons that are thought to facilitate spinal pain transmission may contribute to the reduced opioid analgesia during opioid tolerance.

## Background

Opioid analgesics, such as morphine, currently are the most effective and frequently used pain reliever for moderate to severe pain. However, long-term administration of opioids can alter the central pain-related systems and results in opioid tolerance (decreased analgesic effect of opioids) and opioid dependence (a behavioral state requiring continued opioids to avoid a series of aversive withdrawal syndromes). Opioid tolerance and dependence significantly hamper the effective treatment of chronic pain with opioid analgesics [1]. Numerous agonists and antagonists of various receptors and inhibitors of second messenger pathways have been reported to block or reduce morphine tolerance and/or dependence [2]. It has been well established that glutamate receptors are critical in the development and maintenance of opioid tolerance [3-6]. However, the underlying mechanisms by which glutamate receptors mediate opioid tolerance and dependence remain unclear. An upregulation of the cAMP/PKA signaling pathway has been characterized as a typical molecular adaptation in several brain regions following chronic morphine treatment [1], but the detailed role of the cAMP pathway in analgesic tolerance to chronic opioids has yet to be demonstrated.

Nucleus raphe magnus (NRM), a key medullary relay for descending pain modulation, is critically involved in opioid-induced analgesia [7]. According to their electrophysiological characters and opioid responses, NRM neurons in an *in vitro *preparation have been divided into two general types, *primary cells *that lack the μ-opioid receptor and *secondary cells *that contain the μ receptor [8]. Based on the observation that acute opioids inhibit GABA synaptic transmission in primary cells, we have proposed that opioids produce analgesia in the NRM by disinhibiting or activating those primary cells that send descending projections to the spinal dorsal horn and inhibit spinal pain transmission [8,9]. Several lines of evidence suggests that some NRM cells that are directly inhibited by opioids or contain μ receptors have a facilitating action on spinal pain transmission through their descending projections [7,10,11]. Thus, both activation of pain-inhibiting primary cells and inhibition of pain-facilitating secondary cells in the NRM may be involved in acute opioid-induced analgesia. The synaptic connections between primary cells and secondary cells and the neurotransmitter they release are currently unknown. Accumulating evidence has clearly demonstrated that the μ receptor-containing cells in the NRM are activated in many chronic pain conditions with pain sensitization [11-13], but the activation mechanisms remain unclear. The present study was aimed to investigate chronic morphine-induced adaptation of glutamate synaptic transmission in NRM neurons from morphine-tolerant rats and the intracellular signaling pathways involved in the synaptic adaptation.

## Results

### Chronic morphine selectively increases presynaptic glutamate release

**Figure 1:**
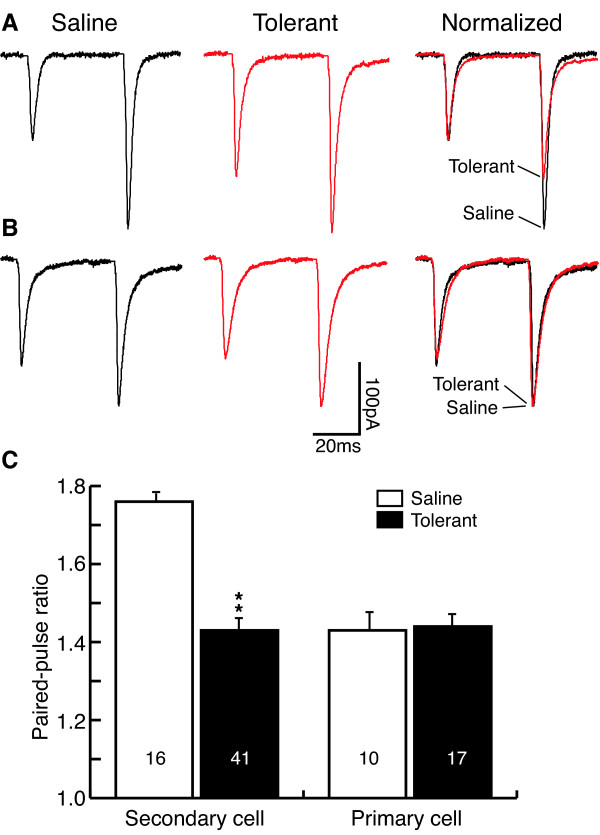
Chronic morphine decreases the paired-pulse ratio (PPR) of glutamate EPSCs in μ-opioid receptor-containing cells (termed secondary cells) in the NRM from morphine-tolerant rats. **A**, Representative EPSC pairs in secondary cells from a saline-treated rat, a morphine-treated tolerant rat and the same two EPSC pairs normalized to the first EPSC. **B**, Representative EPSC pairs in the other type of NRM cells (termed primary cells) lacking the μ receptor from two similar groups of rats. **C**, Group data of the PPR in the two cell types from saline control and morphine-tolerant rats. Stimulus artifacts are blanketed. Numbers in columns indicate cell numbers. ** p < 0.01.

Glutamate-mediated excitatory postsynaptic currents (EPSCs) were recorded under whole-cell voltage-clamp in NRM slices *in vitro*. The EPSCs were compared between NRM slices from saline-treated control rats and those from morphine-treated tolerant rats. Both groups of slices (control and tolerant) were maintained in 5 μM morphine throughout recording experiment *in vitro *to prevent morphine withdrawal (Ingram et al., 1998). A separate group of control slices kept in a morphine-free solution (normal group) was used as controls for the acute morphine added. We used the paired-pulse ratio (PPR) to assess chronic morphine-induced changes in glutamate synaptic transmission in the two types of NRM neurons, primary cells and secondary cells. In the μ receptor-containing secondary cells in control slices, the average PPR was 1.76 ± 0.05 (n = 16), indicating a common synaptic facilitation (PPR>1) by the two stimuli. However, in secondary cells from morphine-tolerant rats, the PPR was significantly smaller than that in controls (1.43 ± 0.07, n = 41, P < 0.01, Fig. 1A,C), indicating an increased probability of presynaptic glutamate release in these secondary cells in a morphine-tolerant state. The PPR in secondary cells in the normal group without 5 μM morphine was 1.89 ± 0.13 (n = 33), which was not statistically different from that in control cells kept in 5 μM morphine (p > 0.05), excluding possible effect of the acute morphine on the PPR. In contrast to secondary cells, there was no significant difference between the EPSC PPRs in μ receptor-lacking primary cells from control and from morphine-tolerant rats (control, 1.43 ± 0.11, n = 10; tolerant, 1.44 ± 0.08, n = 17; P > 0.05, Fig 1B,C). These data indicate that chronic morphine increases glutamate synaptic transmission selectively in the μ receptor-containing secondary cells in a morphine-tolerant state.

### EPSC-enhancing effect of activators of cAMP pathway is increased

**Figure 2:**
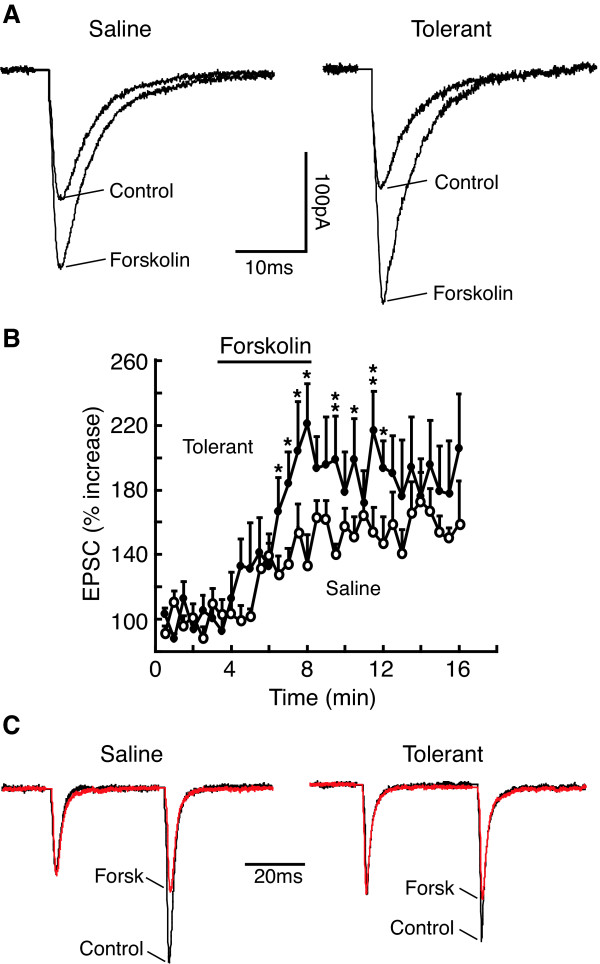
EPSC-enhancing effect of Forskolin is increased in secondary cells from morphine-tolerant rats. **A**, Glutamate EPSCs before (control) and during bath application of forskolin (10 μM) in cells from saline control and tolerant rats. **B**, Time course for the forskolin enhancement of EPSC amplitudes in saline control (n = 12) and tolerant cells (n = 7). The horizontal bar indicates application time for forskolin. **C**, Normalized EPSC pairs in the absence (control) and presence of forskolin in saline control and tolerant cells. Note forskolin-induced decrease in the PPR of both groups. Forsk, forskolin. * p < 0.05, ** p < 0.01.

In control secondary cells, bath application of the adenylyl cyclase (AC) activator forskolin (10 μM) significantly increased the amplitude of evoked EPSCs (eEPSCs) by 53.9 ± 4.1% (control, 95.1 ± 7.3 pA, forskolin, 144.1 ± 14.2 pA, n = 12, P < 0.01). Forskolin produced a comparable EPSC increase in secondary cells in normal slices without 5 μM morphine (57.4 ± 11.4%, n = 6, p < 0.01, p > 0.05 when compared to its effect in control slices with 5 μM morphine). However, in tolerant secondary cells, the EPSC-enhancing effect of forskolin was significantly increased to 95.3 ± 5.3% (p < 0.01, compared to its effect in control group) (control, 88.9 ± 7.8 pA, forskolin, 174.3 ± 13.9 pA, n = 7, P < 0.01, Fig. 2A,B). Similar to the effect of chronic morphine, forskolin (10 μM) also significantly decreased the PPR of glutamate EPSCs in secondary cells from both saline-treated and morphine-tolerant rats (saline: control, 1.73 ± 0.21, forskolin, 1.03 ± 0.14, n = 5, P < 0.01; tolerant: control, 1.43 ± 0.14, forskolin, 0.90 ± 0.03, n = 5, P < 0.01, Fig 2C). Due to a significant decrease in the basal PPR, direct comparison in percentage term of the forskolin effects between the two groups was not feasible. In primary cells, by contrast, although forskolin increased the eEPSC amplitude in both control and tolerant groups, the magnitude of its effects in the two groups was not changed (control, 72.8 ± 17.6%, n = 7, tolerant, 67.0 ± 14.9%, n = 5, P > 0.05).

**Figure 3:**
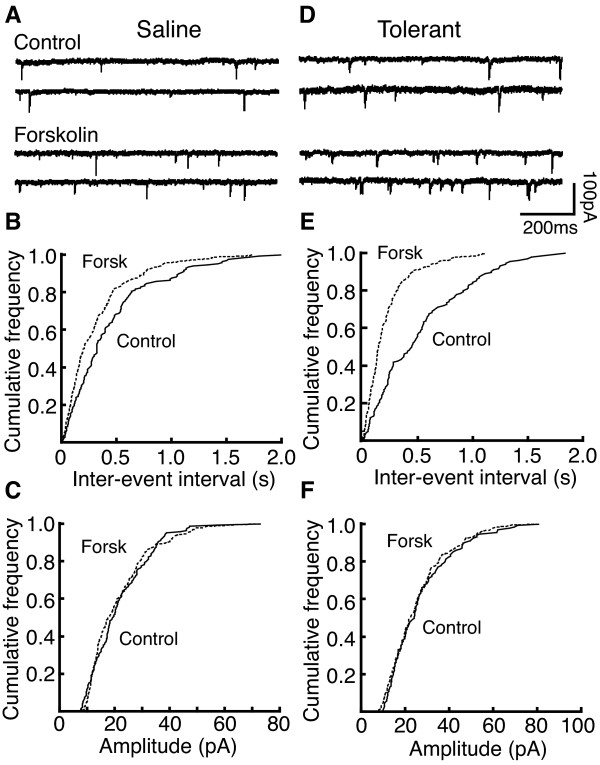
Forskolin enhancement of miniature EPSC (mEPSC) frequency is greater in secondary cells from morphine-tolerant rats. **A**,**B**,**C**, Representative current traces with miniature synaptic events (**A**) and plots of cumulative distribution of mEPSC frequency (**B**) and amplitude (**C**) before and during application of forskolin (10 μM) in the same saline control cell. **D,E,F**, Current traces (**D**) and plots of mEPSC distribution data (**E,F**) from a tolerant cell.

**Figure 4:**
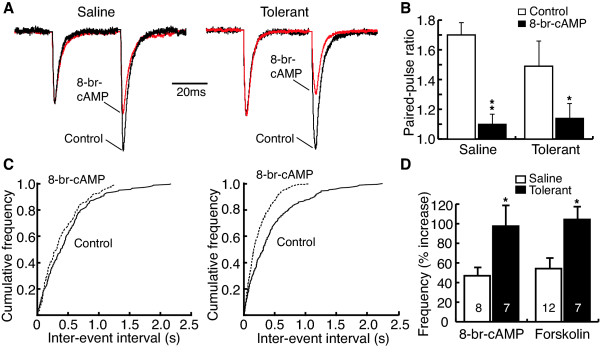
EPSC-enhancing effect of 8-bromo-cAMP is increased in secondary cells from morphine-tolerant rats. **A**, Normalized EPSC pairs in control and in 8-broma-cAMP (8-br-cAMP, 1 mM) in a saline control cell and a tolerant cell. **B**, Group data of 8-br-cAMP-induced decrease in the PPR in saline control (n = 4) and tolerant cells (n = 5). **C**, Distribution data of mEPSC frequency before and during application of 8-br-cAMP in a saline control (left) and a tolerant cell (right). **D**, Percent increase by 8-br-cAMP and forskolin in mEPSC frequency in saline control and tolerant cells.

To further confirm the forskolin effect on presynaptic glutamate release, we examined forskolin action on miniature EPSCs (mEPSCs) in NRM neurons. In control secondary cells, forskolin (10 μM) increased the frequency, but not the amplitude, of glutamate mEPSCs (frequency: control, 5.70 ± 1.03 Hz, forskolin, 8.46 ± 1.07 Hz, n = 5, P < 0.05; amplitude: control, 20.8 ± 2.0 pA, forskolin, 20.6 ± 2.4 pA, n = 5, P > 0.05). This forskolin effect was also observed in secondary cells from tolerant rats (frequency: control, 7.64 ± 0.96 Hz, forskolin, 15.18 ± 0.98 Hz, n = 5, P < 0.01; amplitude: control, 21.3 ± 2.4 pA, forskolin, 20.5 ± 2.23 pA, n = 5, P > 0.05). By comparison, the forskolin-induced increase in the mEPSC frequency was significantly greater in tolerant secondary cells (control, 54.2 ± 9.8%, tolerant, 104.3 ± 11.5%, n = 5, P < 0.05, Fig 3 & Fig. 4D). The forskolin-induced increase in mEPSC frequency in tolerant primary cells was not altered (control, 62.6 ± 17.4%, n = 4, tolerant, 72.2 ± 36.4%, n = 5, P > 0.05).

The cAMP analog 8-bromo-cAMP (8-br-cAMP) produced similar effects to those of forskolin. Bath application of 8-br-cAMP (1 mM) increased the eEPSC amplitude in secondary cells from both saline control and morphine-tolerant rats (saline: control, 110.9 ± 14.8 pA, 8-br-cAMP, 166.6 ± 9.2 pA, n = 4, P < 0.01; tolerant: control, 130.1 ± 1.4 pA, 8-br-cAMP, 260.2 ± 28.5 pA, n = 7, P < 0.001). This cAMP effect was significantly enhanced by chronic morphine treatment (saline, 52.1 ± 9.4%, tolerant, 103.6 ± 10.1%, p < 0.05). 8-br-cAMP also decreased the PPR in secondary cells from both saline control and tolerant rats (saline: control, 1.70 ± 0.08, 8-br-cAMP, 1.10 ± 0.07, n = 4, P < 0.01; tolerant: control, 1.49 ± 0.17, 8-br-cAMP, 1.14 ± 0.10, n = 5, P < 0.05, Fig 4A,B).

Bath application of 8-br-cAMP (1 mM) also significantly increased the frequency of mEPSC in secondary cells from both saline control and tolerant rats (saline: control, 4.72 ± 0.47 Hz, 8-br-cAMP, 6.85 ± 0.70 Hz, n = 8, p < 0.01; tolerant: control, 6.62 ± 0.88 Hz, 8-br-cAMP, 12.46 ± 1.25 Hz, n = 7, p < 0.01, Fig. 4C). However, similar to the forskolin effect, this effect of 8-br-cAMP was also significantly enhanced in the slices from morphine-tolerant rats (saline, 46.9 ± 7.9%, n = 8, tolerant, 97.5 ± 21.2%, n = 7, p < 0.05, Fig. 4D). 8-br-cAMP did not change the amplitudes of mEPSCs in either group (saline: control, 15.9 ± 0.6 pA, 8-br-cAMP, 15.9 ± 1.3 pA, n = 8, P > 0.05; tolerant: control, 18.8 ± 1.2 pA, 8-br-cAMP, 18.9 ± 1.6 pA, n = 7, P > 0.05).

The enhanced forskolin and 8-br-cAMP effects on eEPSC amplitude and mEPSC frequency in morphine-tolerant rats indicate a sensitized or upregulated AC system in a morphine-tolerant state.

### Chronic morphine-induced EPSC increase involves cAMP pathway

**Figure 5:**
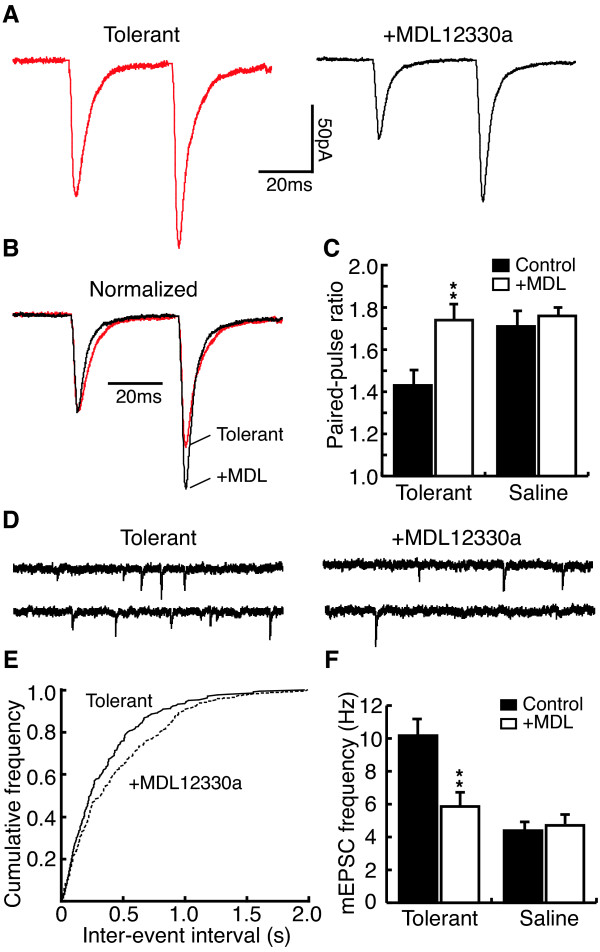
MDL12330a reverses the chronic morphine effect on glutamate EPSCs in tolerant secondary cells. **A**, Representative EPSC pairs in tolerant cells without (tolerant) and after treatment with the adenylyl cyclase (AC) inhibitor MDL12330a (MDL, 100 μM). **B**, Normalized EPSC pairs from **A**. **C**, Group data of the MDL effect on the PPR in cells from morphine-tolerant (n = 16) and saline control rats (n = 5). **D**,**E**, Representative current traces (**D**) and a plot of mEPSC frequency distribution (**E**) in a tolerant cell before and during application of MDL (100 μM). **F**, Group data of the MDL effect on mEPSC frequency in tolerant and saline control cells (n = 5 in each group).

**Figure 6:**
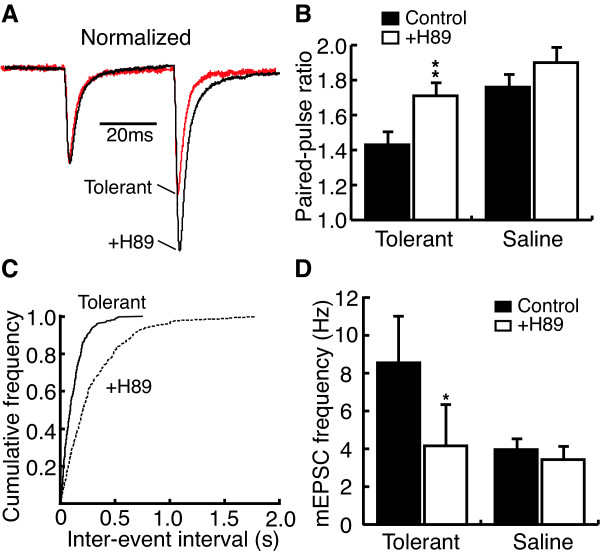
H89 reverses the chronic morphine effect on glutamate EPSCs in tolerant secondary cells. **A**, Normalized EPSC pairs in tolerant cells without and after treatment with H89 (10 μM), a protein kinase A inhibitor. **B**, Group data of the H89 effect on PPR in tolerant (n = 13) and saline control cells (n = 6). **C**, A plot of mEPSC frequency distribution in tolerant cells with or without H89 treatment. **D**, Group data of the H89 effect on mEPSC frequency in tolerant and saline control cells (n = 5 in each group).

To determine the involvement of the AC pathway in the chronic morphine-induced increase of glutamate release in NRM secondary cells, NRM slices were treated with MDL12330a, a selective AC inhibitor. While pre-incubation with MDL12330a (100 μM) did not change the EPSC PPR in secondary cells from control rats (control, 1.71 ± 0.06, MDL, 1.76 ± 0.03, n = 5, p > 0.05), it largely reversed the chronic morphine-induced PPR decrease in secondary cells from morphine-tolerant rats (tolerant, 1.43 ± 0.07, +MDL, 1.74 ± 0.07, n = 16, P < 0.01, Fig. 5A,B,C). Bath application of MDL12330a (100 μM) also significantly decreased the frequency of mEPSCs in tolerant secondary cells (tolerant, 10.17 ± 0.89 Hz, +MDL, 5.86 ± 0.66 Hz, n = 5, P < 0.01, Fig. 5D,E,F) whereas it did not alter the mEPSC amplitude (tolerant, 16.9 ± 1.1 pA, +MDL, 18.1 ± 1.2 pA, n = 5, P > 0.05). MDL had no effect on either the frequency or the amplitude of mEPSCs in secondary cells from saline control rats (frequency: control, 4.39 ± 0.45 Hz, +MDL, 4.71 ± 0.50 Hz, n = 5, P > 0.05; amplitude: control, 16.9 ± 2.0 pA, +MDL, 17.5 ± 1.5 pA, n = 5, P > 0.05, Fig. 5F). Next, we used a PKA inhibitor to determine whether PKA was involved in the enhanced glutamate transmission in secondary cells induced by chronic morphine. Pre-incubation with H89 (10 μM) largely inhibited the chronic morphine-induced decrease of the EPSC PPR in secondary cells from morphine-tolerant rats (tolerant, 1.43 ± 0.07, +H89, 1.71 ± 0.06, n = 13, P < 0.01, Fig. 6A,B). H89 was without effect on the EPSC PPR in secondary cells from control rats (tolerant, 1.76 ± 0.06, +H89, 1.90 ± 0.08, n = 6, P > 0.05, Fig. 6B). Similarly, bath application of H89 (10 μM) significantly decreased the frequencies, but not the amplitude, of mEPSCs in tolerant secondary cells (frequency: tolerant, 8.55 ± 2.42 Hz, +H89, 4.66 ± 2.11 Hz, n = 5, P < 0.05; amplitude: tolerant, 15.1 ± 0.9 pA, +H89, 14.3 ± 0.7 pA, n = 5, P > 0.05, Fig 6C,D). In control secondary cells, H89 did not change either the frequency or the amplitude of mEPSCs (frequency: control, 3.96 ± 0.44 Hz, +H89, 3.43 ± 0.62 Hz, n = 5, P > 0.05; amplitude: control, 20.5 ± 3.8 pA, +H89, 20.2 ± 3.0 pA, n = 5, P > 0.05, Fig. 6D).

These data obtained with both MDL12330a and H89 suggest that the cAMP/PKA pathway is critically involved in the chronic morphine-induced enhancement of glutamate synaptic transmission in NRM secondary cells.

### Chronic morphine-induced EPSC increase involves PKC pathway

**Figure 7:**
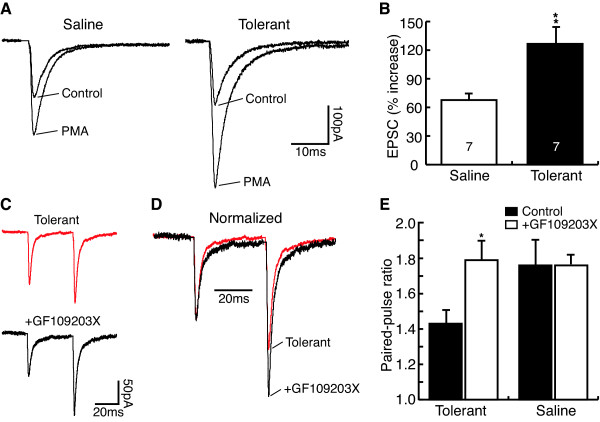
Chronic morphine-induced EPSC augmentation involves the protein kinase C pathway. **A**, Representative EPSCs in the absence and presence of the protein kinase C (PKC) activator phorbol 12-myristate 13-acetate (PMA, 1 μM) in a saline control cell and a tolerant cell. **B**, PMA effect on the peak amplitude of eEPSCs in saline control and tolerance cells. **C**,**D**, Representative EPSC pairs (**C**) and the same pairs after normalization (**D**) in tolerant cells with or without treatment with the PKC inhibitor GF109203X. **E**, Group data of the GF109203X effect on PPR in tolerant (n = 8) and saline control cells (n = 6).

Finally, we examined whether the PKC pathway was also involved in the chronic morphine effect on glutamate EPSCs. Phorbol 12-myristate 13-acetate (PMA, 1 μM), a phorbol ester activator of PKC, increased the amplitude of eEPSCs in NRM secondary cells from both saline control and morphine-tolerant rats (saline: control, 109.9 ± 15.8 pA, PMA, 180.4 ± 22.2 pA, n = 7, P < 0.001; tolerant: control, 134.8 ± 12.9 pA, PMA, 284.7 ± 25.8 pA, n = 7, P < 0.01, Fig. 7A). However, when the PMA effects were compared between the two groups, the EPSC-enhancing effect of PMA was significantly larger in cells from tolerant rats than that in control cells (control, 67.7 ± 6.9%, n = 7, tolerant, 126.6 ± 16.4%, n = 7, p < 0.01, Fig. 7B), indicating a likely upregulated PKC pathway. GF109203X (2 μM), a selective PKC inhibitor, had no significant effect on the EPSC PPR in control secondary cells (control, 1.76 ± 0.14, GF109203X, 1.76 ± 0.05, n = 6, P > 0.05), but it reversed the chronic morphine-induced decrease of the EPSC PPR in secondary cells from morphine-tolerant rats (tolerant, 1.43 ± 0.07, +GF109203X, 1.79 ± 0.10, n = 8, P < 0.05, Fig. 7C,D,E).

## Discussion

The present study has illustrated that chronic morphine increases presynaptic release of glutamate in the μ receptor-containing secondary cells in a morphine-tolerant state. It also provides evidence that both cAMP/PKA and PKC signaling pathways are critically involved in this chronic morphine-induced synaptic adaptation during morphine tolerance.

### Upregulation of the cAMP pathway and glutamate release

In the present study, the effect of chronic morphine on glutamate synaptic transmission was primarily assessed by using the paradigm of EPSC PPR in NRM neurons kept in a tolerant state from morphine-tolerant rats [14]. The PPR has been widely used to determine the involvement of a presynaptic site in the mechanism of neurotransmitter release [15-21]. A change in the PPR is inversely related to the probability of transmitter release and thus, a manipulation that increases the probability of transmitter release results in a reduction in the PPR and vise versa. Since the PPR in a cell under a certain condition is relatively stable, the advantage of using the PPR is to avoid large variance usually present in eEPSC amplitudes and mEPSC frequencies among individual cells, making it possible to compare EPSCs in two separate groups of slices, such as those from saline-treated control rats and those from morphine-tolerant rats. Our current results obtained with the PPR analysis suggest that glutamate synaptic transmission is enhanced through a presynaptic mechanism in NRM secondary cells from morphine-tolerant rats. This conclusion is further supported by the effects of activators and inhibitors of the cAMP/PKA or PKC pathway, which mimicked and reversed the effect of chronic morphine, respectively. It is interesting to note that the chronic morphine-induced EPSC enhancement occurred exclusively in μ receptor-containing secondary cells. This may implicate a functional activation of these μ receptor-expressing NRM cells by glutamate inputs during morphine tolerance (see below). The source of these glutamate inputs is currently unclear.

Presynaptic glutamate release is modulated by many complex and interacting proteins and processes, including components of the cAMP/PKA pathway, in presynaptic terminals [22,23]. It has been demonstrated that *in vitro *application of activators of the cAMP/PKA pathway enhances presynaptic release of glutamate in many types of central neurons [23-27]. *In vivo *administration of chronic morphine induces adaptive hyperactivation of the cAMP/PKA signaling pathway in several brain regions [1,27,28]. The hyperactivated cAMP/PKA pathway induced by chronic morphine may induce a series of cellular adaptations, including enhanced transmitter release. However, cAMP-dependent synaptic adaptation induced by *in vivo *administration of chronic morphine has been reported only in GABAergic synapses in central neurons during morphine withdrawal [21,28,29]. The current study provides direct evidence that chronic morphine enhances glutamate synaptic transmission also in a cAMP-dependent way in NRM neurons from morphine-tolerant rats.

Several observations in the current study support a critical role of an upregulated cAMP/PKA pathway in the enhanced glutamate neurotransmission in secondary cells from morphine-tolerant rats. First, the AC activator and cAMP analog mimic the effect of chronic morphine on EPSC PPR. Second, the potency of cAMP activators in enhancing eEPSC amplitude and mEPSC frequency is significantly augmented in neurons from morphine-tolerant rats. Such an augmented effect has been interpreted as the result of a sensitized or upregulated cAMP pathway [21,27,29]. Finally, inhibitors of both AC and PKA reverse the chronic morphine-induced EPSC enhancement. This effect of AC/PKA inhibitors observed in tolerant cells, but not in control cells, indicates an elevated basal activity of the cAMP/PKA pathway induced by chronic morphine. Such an upregulation of basal and stimulated PKA activity has been shown in other brain areas [1,30].

### Upregulation of the PKC pathway and glutamate release

It has been shown that activation of PKC either by the intracellular messenger diacylglycerol or by phorbol esters produces an enhancement of neurotransmitter release including glutamate release in central neurons [23,31,32]. Activated PKC may phosporylate a number of proteins in nerve terminal and increase neurotransmitter release by calcium influx through voltage-gated calcium channels or by direct effects on exocytosis or on vesicle-recycling pathways [33]. Chronic morphine increases PKC activity in the rat brain and spinal cord [34,35]. The increased PKC activity, which may be mediated through opioid activation of the phospholipase C pathway coupled to opioid receptors, can phosphorylate AC and increase its adaptive responses to chronic opioids [27,30]. In the present study, the PKC activator, similar to activators of the cAMP/PKA pathway, exhibited an augmented potency in enhancing glutamate EPSCs in NRM neurons from morphine-tolerant rats, and the PKC inhibitor reversed the effect of chronic morphine. Although non-specific effects of these PKA and PKC inhibitors cannot be completely ruled out, these data indicate a likely upregulated PKC and PKA activity, which is at least partially responsible for the enhanced glutamate synaptic transmission in NRM secondary cells from morphine-tolerant rats.

### Functional implications in morphine tolerance

Extensive evidence has shown that some NRM cells inhibited by μ opioids have a facilitating action on spinal pain transmission through their descending projections [7,10]. Recent pain research shows that those brainstem cells expressing μ-opioid receptors are commonly activated in various chronic pain conditions, including chronic opioid-induced abnormal pain, and contribute to the sensitized pain or hyperalgesia observed during these conditions [11-13,36]. It remains unclear what mediates the activation of these μ receptor-containing cells in those pain conditions. Data from the current study indicate that excessive activity of glutamate synaptic inputs after chronic exposure to opioids may contribute to the activation of these cells during morphine tolerance. An overall effect of this enhanced glutamate synaptic activity in driving these cells needs to be evaluated by taking into account other synaptic inputs such as GABA synaptic transmission.

It has been proposed that the chronic opioid-induced activation of those presumably pain-facilitating cells and consequently, abnormal pain, constitute part of the mechanisms underlying opioid tolerance characterized by a reduced analgesic effect of opioids [36]. For example, microinjection of lidocaine into the NRM area to inactivate cell activity reverses the tactile allodynia and thermal hyperalgesia induced by prolonged exposure to morphine, and attenuates morphine tolerance [37,38]. A critical role of glutamate receptors in the development of opioid tolerance and dependence has been established [3-6]. The present study illustrates an example of the mechanisms by which glutamate receptors and synapses participate in the development of opioid tolerance. An important role of PKC in opioid tolerance has also been demonstrated in previous studies. For instance, PKC inhibition reduces opioid tolerance [34,35,39,40], and PKCγ mutant mice display alleviated morphine tolerance [41].

In summary, the current study shows that chronic morphine enhances glutamate synaptic transmission in μ receptor-containing NRM neurons. This synaptic adaptation appears to be mediated by an upregulated cAMP/PKA and PKC pathway in morphine-tolerant rats. These results may provide key information for pain therapies aiming at inhibiting those brainstem neurons and their descending pain facilitation, which is responsible for the pain sensitization in several chronic pain conditions.

## Methods

All procedures involving the use of animals conformed to the guidelines set by the University of Texas-MD Anderson Cancer Center Animal Care and Use Committee. *Chronic morphine treatment *Male, Wistar neonatal rats were injected (i.p.) twice daily with morphine solutions for 6 days. The dose of the morphine was 10 mg/kg on the first day, and increased by 5 mg/kg each day to reach a maximum dose of 30 mg/kg on day 5. On day 7, the rats were euthanized for brain slice preparation. Saline was injected at the same volume and schedule in a separate group of rats for controls. Morphine tolerance in these neonatal rats has been described previously [42].

*Whole-cell voltage-clamp recording *The methods for NRM brain slice preparations, visualized whole-cell recordings, cell classification and analysis of glutamate EPSCs have been published previously [15]. The brain of a rat (10–14 days old) was cut in a vibratome in cold (4°C) physiological saline to obtain brainstem slices (220–250 μm thick) containing the NRM. A single slice was submerged in a shallow recording chamber and perfused with preheated (35°C) physiological saline (in mM: NaCl, 126; KCl, 2.5; NaH_2_PO_4_, 1.2; MgCl_2_, 1.2; CaCl_2_, 2.4; glucose, 11; NaHCO_3_, 25, saturated with 95% O_2 _and 5% CO_2_, pH 7.2–7.4). Slices were maintained at around 35°C throughout the recording experiment. Neonatal rats were used for better visualization of neurons in brain slices with an infrared Nomarski microscope. It has been demonstrated that the physiological and pharmacological properties of neurons from these young rats are indistinguishable from those of adult rats[9,15]. Visualized whole-cell voltage-clamp recordings were made from identified neurons held at -60 mV with a glass pipette (resistance 3–5 MΩ) filled with a solution containing (mM): potassium gluconate, 126; NaCl, 10; MgCl_2_, 1; EGTA, 11; Hepes, 10; ATP, 2; GTP, 0.25; pH adjusted to 7.3 with KOH; osmolarity 280–290 mosmol/L. An AxoPatch 1-D amplifier and AxoGraph software 4.7 (Axon Instruments, Inc.) were used for data acquisition and on-line/off-line data analyses. A seal resistance of 2 GΩ or above and an access resistance of 15 MΩ or less were considered acceptable. Series resistance was optimally compensated. The access resistance was monitored throughout the experiment. Electrical stimuli of constant current (0.25 ms, 0.2–0.4 mA) were used to evoke EPSCs with bipolar stimulating electrodes placed lateral (200–400 μm) to the recording pipette within the NRM. The difference in glutamate synaptic transmission between control and morphine-tolerant slices was assessed by the paradigm of paired-pulse ratio (PPR). A pair of EPSCs was evoked by two stimuli with an interval of 40 ms. The PPR was determined by dividing the second EPSC amplitude by the first one. Miniature EPSCs were obtained in 60-sec epochs in the presence of tetrodotoxin (1 μM). The AxoGraph software was used to detect and measure the amplitude and intervals of the synaptic events, and to analyze their distribution data. All NRM cells recorded were classified into either a μ-containing secondary cell or a μ-lacking primary cell according to the criteria described in our previous study [8]. NRM slices from both morphine- and saline-treated rats were kept in 5 μM morphine throughout the recording experiment to maintain the slices in a morphine-tolerant state and prevent withdrawal [14]. Another group of slices from saline-treated rats kept in a morphine-free solution was used as controls for the acute morphine. Statistic analysis of mEPSCs were performed with the Mann-Whitney *U *test or the Kolmogorov-Smirnov test using the Statview software. Other numeral data were statistically analyzed with Students' t tests and presented as mean ± S.E.M. Some slices were incubated in MDL12330a (100 μM), or H89 (10 μM) or GF109203X (2 μM) for at least 1 hour. Drugs were generally applied through the bath solution unless otherwise specified. Morphine sulfate was kindly supplied by the National Institute on Drug Abuse. All other drugs were purchased either from Sigma-Aldrich Co. or from Tocris Cookson Inc. (Ellisville, MO).

## Competing interests

The author(s) declare that they have no competing interests.
